# Age‐ and Gender‐Specific Epidemiologic Characteristics of Major Intra‐Articular Fractures: Five‐Year Data from a Level 1 Trauma Center

**DOI:** 10.1111/os.12937

**Published:** 2021-03-30

**Authors:** Wei‐guang Zhao, Yan‐bin Zhu, Jiang‐tao Ma, Xiao‐li Yan, Ying‐ze Zhang

**Affiliations:** ^1^ Department of Orthopedic Surgery HanDan Central Hospital HanDan China; ^2^ Department of Trauma Emergency Center The Third Hospital of Hebei Medical University, Orthopaedics Research Institution of Hebei Province, Key Laboratory of Biomechanics of Hebei Province Shijiazhuang China

**Keywords:** Age distribution, Epidemiology, Intra‐articular fracture, Sex distribution

## Abstract

**Objective:**

To investigate the epidemiological characteristics of major intra‐articular fractures.

**Methods:**

This retrospective study enrolled patients with major intra‐articular fractures who were treated in the Third Hospital of Hebei Medical University from January 2015 to December 2019. A total of 11,084 patients (7,338 [66.20%] males and 3,746 [33.80%] females) meeting the inclusion and exclusion criteria were included. The distribution characteristics of intra‐articular fractures involving shoulder, elbow, wrist, hip, knee, ankle, and subtalar joints were identified.The potential associations between fractures and various other factors, such as age, gender, sites, were explored.

**Results:**

There were 74 cases (0.67%) of shoulder fractures, 1,941 cases (17.51%) of elbow fractures, 1,155 cases (10.42%) of wrist fractures, 520 cases (4.69%) of hip fractures, 3,118 cases (28.13%) of knee fractures, 2,156 cases (19.45%) of ankle fractures, and 2,120 cases (19.13%) of subtalar fractures. The overall male‐to‐female ratio was 1.96:1. The highest proportion age group of major intra‐articular fractures included the ages 45–54 years. For males, the highest proportion age group was 45–54 years, for females, it was 55–64 years. The knee joint fracture was the most common type, accounting for 28.13%. For male and female patients, knee fractures accounted for 26.19% and 31.93%, respectively, with a male to female ratio of 1.13:1. The proportion of shoulder fractures was the smallest among this investigation, accounting for 0.67%. For male and female patients, shoulder fractures accounted for 0.44% and 1.12%, respectively, with a male to female ratio of 0.76:1. The age group with the highest proportion of shoulder joint fractures was ≥65 year olds (41.89%), with a male to female ratio of 0.76:1. The age group with the highest risk of elbow, wrist, hip, knee, ankle, and subtalar joint fracture was 5–14 year olds (33.59%) with a male to female ratio of 3.29:1, 5–14 year olds (23.98%) with a male to female ratio of 6.91:1, 45–54 year olds (26.92%) with a male to female ratio of 5.67:1, 45–54 year olds (24.60%) with a male to female ratio of 1.68:1, 25–34 year olds (20.36%) with a male to female ratio of 2.30:1, 45–54 year olds (27.41%) with a male to female ratio of 9.02:1, respectively. The most common site of intra‐articular fractures in different age groups was corresponding as follows: 0–4 year olds (elbow), 5–14 year olds (elbow), 15–24 year olds (ankle), 25–34 year olds (subtalar joint), 35–44 year olds (subtalar joint), 45–54 year olds (knee), 55–64 year olds (knee), 65–74 year olds (knee), and ≥75 year olds (knee).

**Conclusion:**

The current study revealed the age‐ and gender‐specific epidemiological characteristics of major intra‐articular fractures, providing a basis for clinical evaluation and practices.

## Graphical abstract

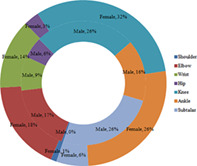

The current study revealed the age‐ and gender‐specific epidemiological characteristics of 11,084 major intra‐articular fractures, providing a basis for clinical evaluation and practices.

## Introduction

Intra‐articular fracture refers to a fracture involving the articular surface, which belongs to a common type of fracture in clinical practice. It is mostly caused by high‐energy injuries or falling injuries, and the treatment requires a high standard of expertise; otherwise, improper handling often creates functional obstacles of different levels. At present, most studies focus on conservative or surgical treatment, and there remains a lack of large‐sample epidemiological investigation; furthermore, a substantial proportion of epidemiological studies focus on a specific location (e.g. knee joint fracture), rather than investigating the epidemiological characteristics of major intra‐articular fractures in the whole body.

As early as 1832, Astley Cooper^1^ recognized the influence of age on bone strength in the study of femoral neck fractures in elderly patients. In 1882, Bruns^2^ first discussed the influence of age and gender on the incidence of various types of fractures. Alffram^3^ studied the age and gender differences of 2672 cases of forearm fractures that occurred in 5 years and found that before the age of 40, the incidence of distal forearm fractures in males and females was approximately equal, while after the age of 60, the number of female patients with fractures was more than seven times that of males. Singer^2^ conducted an epidemiological survey on 15,000 adult fracture patients in the Edinburgh area, and found that between 15 and 49 years old, males were 2.9 times more likely to have fractures than females, while after 60 years old, the situation was reversed, with females 2.3 times more likely to have fractures than males. The influence of age and gender on the incidence and location of fractures also exists in the adolescent population. An epidemiological survey of 6% of the population of Britain^4^ showed that the incidence of fractures in adolescent boys was higher than that in girls, and the peak time was 14 and 11 years old, respectively, with significant differences between them.

Studies have shown that age and gender are closely related to intra‐articular fractures at specific sites^5^, ^6^, ^7^, ^8^. Therefore, it is necessary to conduct relevant epidemiological investigation into these factors to identify the aggregation of intra‐articular fractures at different ages and genders, so as to provide effective targeted preventive measures. Therefore, we conducted this retrospective study on the clinical data of 11,084 patients with major intra‐articular fractures with the aim to clarify: (i) which joint of intra‐articular fracture is most common and the incidence of other intra‐articular fractures; (ii) which age group is most likely to experience the major intra‐articular fractures; and (iii) the distribution of major intra‐articular fractures in different age groups.

## Patients and Methods

### Patients Inclusion

Patients with major intra‐articular fractures of the limbs were selected to be included in this study.

Patients were sourced through the picture archiving and communication systems (PACS) and the medical record inquiry systems of the Third Hospital of Hebei Medical University from January 2015 to December 2019.

Inclusion criteria were as follows: (i) shoulder joint: humeral head, humeral anatomic neck and glenoid; (ii) elbow joint: humeral intercondylar, internal and external condyle of humerus, capitellum, radius head, ulan olecranon, coronal process; (iii) wrist joint: distal radial articular surface; (iv) hip joint: femoral head, acetabulum; (v) knee joint: femoral intercondylar, Hoffo fracture, tibial plateau, patella; (vi) ankle joint: medial malleolus, lateral malleolus, posterior malleolus; (vii) subtalar joint: calcaneus and talus.

Exclusion criteria: (i) patients with pathological fractures; (ii)patients with old fractures.

### Demographic Information and Fracture Classification

The medical records of enrolled patients were retrieved using the medical record inquiry system. The demographic characteristics of patients and detailed information on major intra‐articular fractures were recorded. The preoperative radiographs, computed tomography (CT), and magnetic resonance imaging (MRI) of the intra‐articular fractures were collected with the use of the picture archiving and communication systems. The X‐ray films, CT, and MRI scans of the intra‐articular fractures were reviewed by five orthopaedic surgeons with more than 10 years of experience. Intra‐articular fractures are divided into shoulder intra‐articular fractures, elbow intra‐articular fractures, wrist intra‐articular fractures, hip intra‐articular fractures, knee intra‐articular fractures, ankle intra‐articular, and subtalar intra‐articular fractures according to the fracture site. If there was any disagreement in the diagnosis of intra‐articular fractures, a final decision would be made through discussion, with consensus achieved by at least three surgeons.

### Age Groups

There were nine groups stratified by age: 0–4 years, 5–14 years, 15–24 years, 25–34 years, 35–44 years, 45–54 years, 55–64 years, 65–74 years, and ≥ 75 years.

### Indicators Measures

An intra‐articular fracture is a fracture that crosses a joint surface, so all the patients we included met the definition of intra‐articular fracture. Preoperative X‐ray, CT, and MRI were used for auxiliary diagnosis of intra‐articular fractures. Intra‐articular fractures in seven major sites of the human body were selected for analysis and each intra‐articular fracture included the following fracture type:

#### Intra‐articular Fractures of Shoulder

Intra‐articular fractures of shoulder include humeral head, humeral anatomic neck and glenoid.

#### Intra‐articular Fractures of Elbow

Intra‐articular fractures of elbow include humeral intercondylar, internal and external condyle of humerus, capitellum, radius head, ulan olecranon, coronal process.

#### Intra‐articular Fractures of Wrist

Intra‐articular fractures of wrist include distal radial articular surface.

#### Intra‐articular Fractures of Hip

Intra‐articular fractures of hip include femoral head, acetabulum.

#### Intra‐articular Fractures of Knee

Intra‐articular fractures of knee include femoral intercondylar, Hoffo fracture, tibial plateau, patella.

#### Intra‐articular Fractures of Ankle

Intra‐articular fractures of ankle include medial malleolus, lateral malleolus, posterior malleolus.

#### Intra‐articular Fractures of Subtalar joint

Intra‐articular fractures of subtalar joint include calcaneus and talus.

### Statistical Analysis

All the analyses were performed with the use of SPSS 22.0 (IBM, Chicago,USA). A *P*‐value of <0.05 was considered significant. Descriptive data were presented as numbers. The number of major intra‐articular fractures and composition ratios in each year were analyzed. The distribution of male and female patients in different age groups and the distribution of intra‐articular fractures in different age groups were all accessed. The Kolmogorov–Smirnov test was used to check whether results were in accordance with the normal distribution. The compositions of intra‐articular fractures in different years were analyzed by Analysis of Variance (ANOVA) test, and the comparison between pairs was performed by Student‐Newman‐Keuls Q test (SNK‐Q test) or Least Significant Difference test (LSD test). The compositions of intra‐articular fractures in different genders over 5 five years were compared by Pearson chi‐square test.

## Result

### Demographic Information and Fracture Classification

**Fig. 1:**
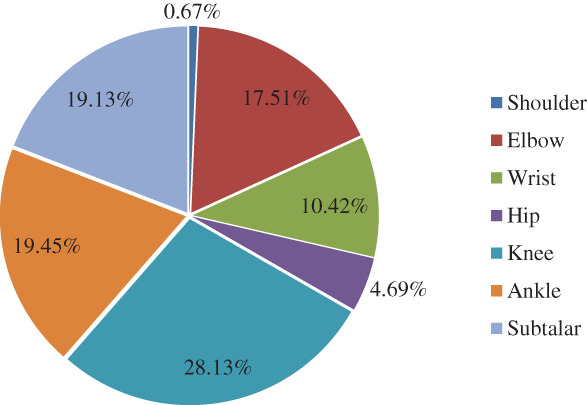
Distribution of different intra‐articular fractures in 11,084 patients during the 5‐year period from a level 1 trauma center. The incidence of these fractures from high to low was: knee (3118 cases), ankle (2156 cases), subtalar (2120 cases), elbow (1941 cases), wrist (1155 cases), hip (520 cases), shoulder (74 cases).

**Fig. 2:**
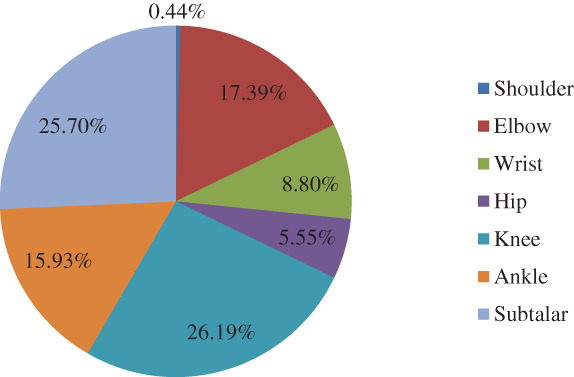
Distribution of the seven intra‐articular fractures in male patients during the 5 years, showing that knee fractures were the most common and shoulder fractures were the least common, accounting for 26.19% and 0.44% respectively.

**Fig. 3:**
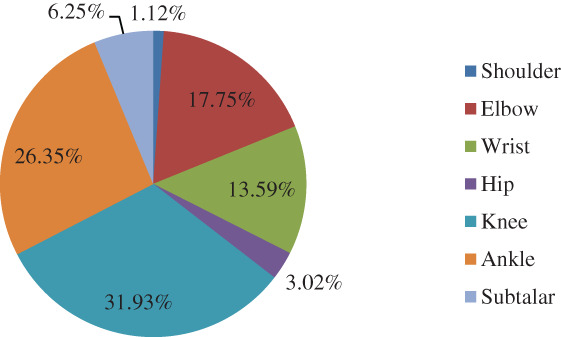
Distribution of different intra‐articular fractures in female patients, showing that knee fractures were also the most common, and at an even higher rate compared to males.

A total of 11,084 patients with major joint fractures were identified over the 5‐year period, accounting for 17.08% (11084/64885) of the total fractures at the whole skeleton sites in the same period, including 7338 males and 3746 females. The overall male‐to‐female ratio was 1.96:1. The highest proportion age group was 45–54 years (20.49%), the male and female patients were 45–54 years (20.89%) and 55~64 years (23.63%), respectively. There were 74 cases (0.67%) of shoulder fractures, 1,941 cases (17.51%) of elbow fractures, 1,155 cases (10.42%) of wrist fractures, 520 cases (4.69%) of hip fractures, 3,118 cases (28.13%) of knee fractures, 2,156 cases (19.45%) of ankle fractures, and 2,120 cases (19.13%) of subtalar fractures. From 2015 to 2019, the numbers of patients with intra‐articular fractures was 1992, 2039, 2118, 2474, and 2461, showing an increasing trend year by year. There was no significant difference in the composition of intra‐articular fractures over the 5 years (*P* > 0.05), and there was a significant difference in the composition of intra‐articular fractures between men and women (χ2 = 764.06, *P =* 0.000). The proportion of intra‐articular fractures in each year and in different genders is shown in Tables 1 and 2 and Figs 1, 2, 3.

**TABLE 1:** Gender distribution of 11,084 intra‐articular fractures from 2015 to 2019

Joint	2015			2016			2017			2018			2019			Sum
	Male	Female	Sum	Male	Female	Sum	Male	Female	Sum	Male	Female	Sum	Male	Female	Sum	
Shoulder	9	13	22	3	7	10	11	8	19	2	2	4	7	12	19	74
Elbow	205	83	288	259	132	391	256	139	395	259	136	395	297	175	472	1941
Wrist	75	54	129	71	34	105	65	31	96	292	233	525	143	157	300	1155
Hip	73	19	92	63	16	79	70	24	94	92	20	112	109	34	143	520
Knee	394	242	636	430	298	728	396	197	593	362	200	562	340	259	599	3118
Ankle	243	216	459	187	176	363	284	210	494	258	212	470	197	173	370	2156
Subtalar	314	52	366	333	30	363	385	42	427	369	37	406	485	73	558	2120
Sum	1313	679	1992	1346	693	2039	1467	651	2118	1634	840	2474	1578	883	2461	11084

**TABLE 2:** Distribution of intra‐articular fractures in male and female patients

Joint	Male	Female
Shoulder	32 (0.44%)	42 (1.12%)
Elbow	1276 (17.39%)	665 (17.75%)
Wrist	646 (8.80%)	509 (13.59%)
Hip	407 (5.55%)	113 (3.02%)
Knee	1922 (26.19%)	1196 (31.93%)
Ankle	1169 (15.93%)	987 (26.35%)
Subtalar	1886 (25.70%)	234 (6.25%)
Sum	7338	3746

### The Most Affected Age Group and Its Gender Distribution for Each Intra‐articular Fracture

The knee fractures were the most common among the seven intra‐articular fractures, accounting for 28.13%, and the ratio of males to females was 1.13:1. The least common fracture was shoulder fractures, accounting for 0.67%, 0.44% in male and 1.12% in female, with a male to female ratio of 0.76:1. The age group with the highest proportion of shoulder fractures were ≥65 year olds (41.89%), with a male to female ratio of 0.76:1. The peak incidence occurred at 5–14 years of age (33.59%) in elbow fractures, with a male to female ratio of 3.29:1. For other joints, such as wrist, hip, knee, ankle, and subtalar joint, the age groups with the highest proportion of fractures were 5–14 years (23.98%), 45–54 years (26.92%), 45–54 years (24.60%), 25–34 years (20.36%), 45–54 years (27.41%), respectively, and the male‐to‐female ratio was 6.91:1, 5.67:1, 1.68:1, 2.30:1, and 9.02:1, respectively.

### The Most Common Intra‐articular Fractures in Different Age Groups

**Fig. 4:**
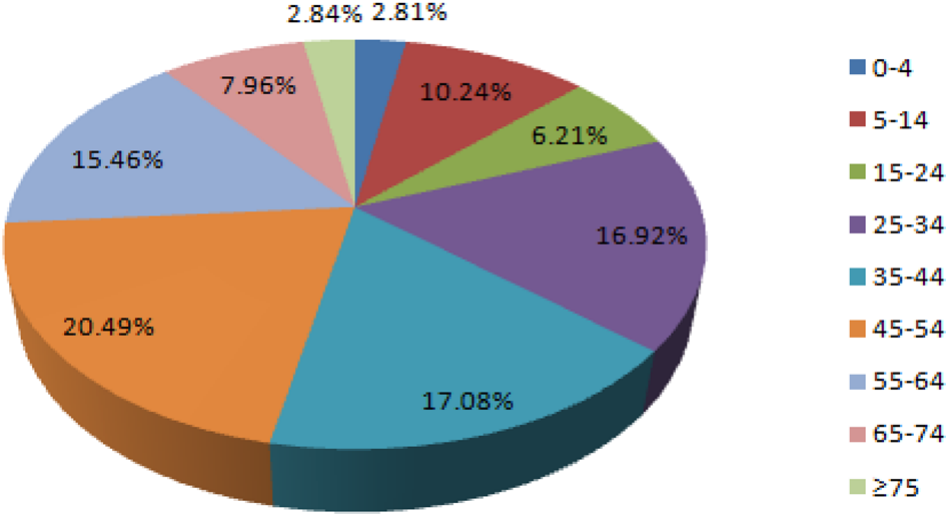
The proportion of intra‐articular fractures in different age groups, showing that the highest proportion age group was 45–54 years (20.49%). While 0–4 years rarely experienced intra‐articular fractures.

**Fig. 5:**
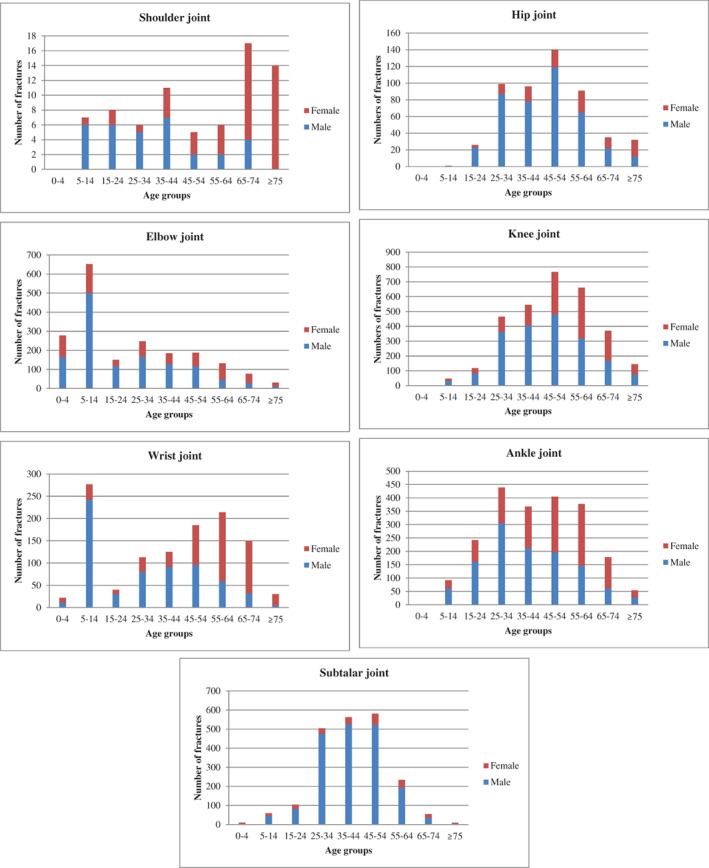
Age‐ and gender‐specific number of fractures in selected joints among 11,084 men and women treated in the Third Affiliated Hospital to Hebei Medical University, 2015–2019.

The most common intra‐articular fracture sites in different age groups were: 0–4 years (elbow, 89.39%); 5–14 years (elbow, 57.44%); 15–24 years (ankle, 35.17%); 25–34 years (subtalar, 26.93%); 35–44 years (subtalar, 29.74%); 45–54 years (knee, 33.77%); 55–64 years (knee, 38.51%); 65–74 years (knee, 42.06%); ≥75 years (knee, 46.03%). There were significant differences in the composition of intra‐articular fractures among different age groups (*P* = 0.000), as shown in Table 3 and Fig. 4. Age‐ and gender‐specific numbers of fractures at selected joints among 11,084 men and women is shown in Fig. 5.

**TABLE 3:** Distribution of intra‐articular fractures in different age groups and genders

Age	Shoulder		Elbow		Wrist		Hip		Knee		Ankle		Subtalar		Sum
	Male	Female	Male	Female	Male	Female	Male	Female	Male	Female	Male	Female	Male	Female	
0–4	0	0	165	113	11	11	0	0	0	0	0	1	3	7	311
5–14	6	1	500	152	242	35	1	0	32	15	60	32	45	14	1135
15–24	6	2	117	33	30	10	23	3	81	37	161	81	82	22	688
25–34	5	1	167	81	80	33	87	12	362	103	306	133	475	30	1875
35–44	7	4	127	58	91	34	78	18	405	140	212	156	526	37	1893
45–54	2	3	116	72	96	89	119	21	481	286	196	209	523	58	2271
55–64	2	4	47	85	59	155	65	26	317	343	146	231	193	41	1714
65–74	4	13	27	50	32	117	22	13	168	203	61	117	35	20	882
≥75	0	14	10	21	5	25	12	20	76	69	27	27	4	5	315
Sum	32	42	1276	665	646	509	407	113	1922	1196	1169	987	1886	234	11084

## Discussion

Intra‐articular fracture is a common type of injury in trauma and orthopaedics. The principle of treatment requires precise reduction of the articular surface; improper treatment can likely result in postoperative complications, such as pain and dysfunction, which will increase the burden on society and family. At present, more attention has been paid to the treatment of intra‐articular fractures in clinic, but less to primary prevention. Furthermore, existing epidemiological studies are mostly concerning with the individual intra‐articular fractures and there are only rare epidemiological holistic studies on whole major intra‐articular fractures. Although this is not a national investigation study, it includes a large number of cases over a long time span. For the first time, this study elaborates on age and gender as factors that influence different intra‐articular fractures, making it a valuable reference for the prevention of these types of fractures.

### Overall Age and Sex Distribution Characteristics of Major Intra‐articular Fractures in the Human Body

Male patients were about twice as likely as females to experience these fractures, possibly because they offen participate in sports or jobs that were vulnerable to injury. A total of 3118 patients with intra‐articular knee fractures were treated in the past 5 years, accounting for 28.13% of fractures, making them the most common intra‐articular fractures. The reason may the high proportion of patellar and tibial plateau fractures, with incidence rates of 2.73%^9^ and 1.66%^10^, respectively. Previous studies showed that patellar fractures and tibial plateau fractures were more common in males^9^, ^10^, and the highest proportion age group was 41–59 years, which was basically consistent with the findings of our study. A study^11^ based on 576,364 patients in the northern region of Denmark showed that the incidence of tibial plateau fractures was 10.3 per 100,000, and the highest frequency was in 40–60 year olds for both men and women. At the same time, that study pointed out that fracture rates were higher in men under age 50, but after age 50, fracture rates increased significantly in women and decreased in men. The main causes of injury for men were falls from a height or accidents involving cars, motorcycles, or other motorized vehicles. Women were injured primarily while bicycling, walking, and during indoor activities. This indicated that close attention should be paid to the prevention of knee fractures in this age group, such as avoiding falls, traffic injuries, preventing osteoporosis, and so on.

### Age and Gender Characteristics of Intra‐articular Fractures at Different Sites

This study showed that the age group with the highest proportion of shoulder fractures was ≥65 year olds (41.89%) and the male‐to‐female ratio was 0.76:1, which was the only age group where the number of female patients exceeded that of male patients. The reason may be that osteoporosis of females was more severe in older women than in older men^12^, ^13^, ^14^. A Finnish study^15^ showed that age and gender were both independent risk factors in patients over the age of 60 with proximal humerus fractures caused by low energy.

The age group at risk of elbow and wrist joint fractures was 5–14 year olds, and males were significantly more prone to these fractures than females, which was related to the lively and active character of boys in this age group^16^. Therefore, attention should be paid to popularizing relevant sport protection knowledge, wearing protective gear, etc. The findings are consistent with the investigation by Erik^17^, who conducted an epidemiological survey of fractures in children and adolescents in Sweden during the past 10 years, showing that the most common fracture site was the distal forearm, the peak incidence occurring at 11~12 years in females and at 13 ~ 14 years in males. The most common risk factors were various sports.

The age group with the highest proportion of hip, knee, and subtalar joint fractures was 45–54 year olds, with more males than females experiencing these fractures. Obviously, this age group includes first‐home owners. Therefore, it is necessary to strengthen the protection of lower limb joints in this age group. This study also showed that peak incidences of ankle fractures occurred at 25–34 years, especially in men, suggesting that attention should be paid to the protection of ankle joints in young men who engage in more construction and transportation activities. Similar to previous studies, Liu^14^ retrospectively analyzed the data of 7742 ankle fractures and concluded that the highest‐proportion age group was 21–30 year olds, with a male‐to‐female incidence ratio of 1.36. However, a nationwide epidemiological survey of ankle fractures based on the basic population by Liu^9^ showed that the peak incidence occurred at 41~50 years, which was different from our study because of the different study population. Another^18^ epidemiological survey of ankle fractures in 673,214 cases in American hospitals showed that the highest incidence age group was 10–19 year olds (23.5%), followed by 20–29 year olds (12.0%), with females (56%) more likely to experience these fractures than males (44%). The common causes were falls and sports injuries, and occurred more usually in white people than other races. The difference between the two studies was possibly that ours was a single‐center study, while the American study was a multi‐center study. In future research, the sample should be expanded and multi‐center data should be included.

### Distribution Characteristics of Intra‐articular Fractures in Different Age Groups

The elbow fractures were the most common in 0–4 year olds and 5–14 year olds, which may be related to more activity patterns and high incidences of humeral condyle and olecranon^19^ in this age group. The results are consistent with another investigation^20^, which included 8987 fractures over a 5‐year period, showing that school‐age patients aged 7–11 years accounted for the most fractures (32.86%), and the most common fracture site was the distal humerus (22.4%). Intra‐articular fracture of the ankle was the most common in 15–24 year olds, followed by elbow fractures. While intra‐articular fractures of the subtalar joints were common in 25–44 year olds, the reason may be that the age groups were young adults, belonging to the main labor force in society, which exposed them to the risk of high‐energy trauma, such as crashes and falls from height, resulting in calcaneal and talus fractures. The epidemiological study, which included 3881 calcaneal fractures, showed that the age group with the highest propotion was 31–40 year olds ^21^. Dong^22^ analyzed 1014 cases of talar fracture, showing that the peak incidence occurred at 21–40 years, and the median age at 35–36 years. Knee fractures were most common in patients over 45 years of age, as the incidence of osteoporotic fractures around the knee increased with age^23^.

By dividing patients into age groups, we defined the most common intra‐articular fracture sites in each age group, which would be helpful to institute preventive and protective measures in order to reduce the incidence of intra‐articular fractures and avoid complications such as deformity, dysfunction, and pain. Finally, the burden on both families and society will be reduced.

### Limitations

Several limitations of this study should be noted. First, because it was a retrospective study, recall bias is inevitable. Second, the cases were only from a single center and other factors such as mechanism of injury, occupation, classification of fractures were not taken into account. Therefore, the next step is to expand the sample size through a multi‐center study and consider more related factors.

### Conclusion

The most common fracture site among major intra‐articular fractures is in the knee, while the least common major intra‐articular fracture site is the shoulder. Male patients are more likely to experience these fractures than females. At 0–4 years and 5–14 years, the most common intra‐articular fractures occur in the elbow. However, ankle fractures, subtalar fractures, and knee fractures are most common in 15–24 year olds, 25–44 year olds, and >45 year olds, respectively. Targeted preventive measures can be formulated according to the above characteristics.

## Author Contributors

Yingze Zhang conceived the idea for the study; Weiguang Zhao designed the study. Yanbin Zhu, Jiangtao MA, Xiaoli Yan collected the relevant data. Yanbin Zhu and Jiangtao Ma prepared the tables and figures. Weiguang Zhao performed the statistical analyses. All the authors interpreted the data and contributed to preparation of the manuscript.
