# Corrigendum

**DOI:** 10.1111/os.13141

**Published:** 2021-07-26

In [1], Wei‐guang Zhao's first affiliation was omitted in error. To correct this, the order of the affiliations have to be adjusted and all authors' affiliations have to be accordingly renumbered.

The authors' names and the numerals linking their affiliations should thus appear as follows in the author by‐line:

Wei‐guang Zhao^1,2^, Yan‐bin Zhu^1^, Jiang‐tao Ma^1^, Xiao‐li Yan^1^, Ying‐ze Zhang^1^.

The reordered and renumbered affiliations should appear as follows:

^1^Department of Trauma Emergency Center, The Third Hospital of Hebei Medical University, Orthopaedics Research Institute of Hebei Province, Key Laboratory of Biomechanics of Hebei Province, Shijiazhuang and ^2^Department of Orthopaedic Surgery, HanDan Central Hospital, HanDan, China.

The authors apologize for the error and any inconvenience it may have caused.
